# Reinforced tension-line suture after laparotomy: long-term results of Rein4CeTo1 randomized clinical trial

**DOI:** 10.1093/bjsopen/zraf150

**Published:** 2026-01-01

**Authors:** Charlotta L Wenzelberg, Peder Rogmark, Olle Ekberg, Ulf Petersson, Carl-Fredrik Rönnow

**Affiliations:** Department of Clinical Sciences Malmö, Lund University, Lund, Sweden; Department of Surgery, Skåne University Hospital Malmö, Malmö, Sweden; Department of Clinical Sciences Malmö, Lund University, Lund, Sweden; Department of Surgery, Skåne University Hospital Malmö, Malmö, Sweden; Department of Translational Medicine Malmö, Lund University, Lund, Sweden; Department of Radiology Diagnostics, Skåne University Hospital Malmö, Malmö, Sweden; Department of Clinical Sciences Malmö, Lund University, Lund, Sweden; Department of Surgery, Skåne University Hospital Malmö, Malmö, Sweden; Department of Clinical Sciences Malmö, Lund University, Lund, Sweden; Department of Surgery, Skåne University Hospital Malmö, Malmö, Sweden

## Abstract

**Background:**

Incisional hernia remains the most common complication of open abdominal surgery. The aim was to investigate whether a reinforced tension-line suture combined with standard 4 : 1 small-bite closure reduces the 3-year incidence of computed tomography-detected incisional hernia in open colorectal cancer surgery.

**Methods:**

Patients aged > 18 years, scheduled for colorectal cancer resection through a midline incision between 2017 and 2021 at Skåne University Hospital Malmö and Kristianstad County Hospital, Sweden, were eligible for inclusion. Patients were randomized to fascial closure by reinforced tension-line suture combined with 4 : 1 small-bite closure with polypropylene sutures (RTL group) or 4 : 1 small-bite closure alone with polydioxanone sutures (PDS group), in a 1 : 1 ratio. Computed tomography interpreters were blinded to study groups. Univariate, bivariate, and multivariate logistic regression analyses were performed to investigate and adjust study groups for potential risk factors for incisional hernia.

**Results:**

The study randomized 80 patients in each group. At 3 years, 101 remained for analysis: 43 in the RTL group and 58 in the PDS group. Incisional hernia was detected in 27 patients: 6 of 43 (14%) in the RTL and 21 of 58 (36%) in the PDS group, resulting in a significant risk difference of 22% (odds ratio 3.50, 95% confidence interval 1.27 to 9.66; *P* = 0.016). In multivariate analysis, the PDS group (odds ratio 3.40, 1.14 to 10.14; *P* = 0.028) and adjuvant chemotherapy (odds ratio 2.98, 1.10 to 8.08; *P* = 0.032) were significant risk factors for incisional hernia. No adverse events related to the closure techniques were found in either group.

**Conclusion:**

Adding a reinforced tension-line suture significantly reduced the long-term incidence of incisional hernia compared with the 4 : 1 small-bite technique alone in patients undergoing open colorectal cancer surgery. These findings suggest that the reinforced tension-line suture is an efficient and easy way to prevent incisional hernia.

## Teaser

The aim of this randomized clinical trial was to investigate whether a reinforced tension-line (RTL) suture reduces 3-year computed tomography-detected incisional hernia incidence. The RTL technique significantly reduced the incidence of incisional hernia compared with the standard facial closure technique in patients undergoing open colorectal cancer surgery. The incisional hernia incidence was 6 of 43 (14%) in the RTL and 21 of 58 (36%) in the standard facial closure group.

## Introduction

Incisional hernia (IH) constitutes the most common long-term complication associated with open abdominal surgery^1–5^. The incidence of IH is uncertain, ranging from 0 to 36% in a large meta-analysis^6^, but rates have been reported to be as high as 60% in specific high-risk populations^3,6^. Minimally invasive techniques have reduced the proportion of patients undergoing midline laparotomy, but open abdominal surgery will continue to be performed to some extent in the foreseeable future. In addition, minimally invasive techniques often require a restricted midline incision for specimen extraction with a non-negligible risk of IH^7^.

The clinical presentation and symptoms of IH vary greatly, from absence of symptoms to pain, discomfort, and impaired quality of life, to potentially life-threatening disorders including incarceration and bowel strangulation^1,5^. Besides patient-related outcomes, IH has a substantial economic impact on healthcare costs, estimated to be > $3 billion annually in the USA in 2006^8,9^. Consequently, great efforts have been made to reduce the incidence of IH in past decades with use of different fascial closure techniques.

The small-bite 4 : 1 technique, introduced by Milbourn *et al.*^10^, is currently the standard and recommended in the European Hernia Society (EHS) guidelines^11^. Discouragingly, IH is still a common complication even with the 4 : 1 small-bite technique, with a reported rate of 13% at 1 year after surgery^12^. On the other hand, prophylactic mesh, which has been shown to decrease IH rates in high-risk patients, has not been implemented widely owing to fear of complications, increased costs and time consumption^13–16^. Thus, novel ways to reduce the incidence of IH, without substantially increasing operating time, costs, and risk of complications, are warranted.

Notably, the reinforced tension-line (RTL) suture, comprising a longitudinal suture parallel to the edge of the fascial incision, was initially described in 2007 by Hollinsky *et al.*^17^. The technique was proposed as an alternative to mesh repair in patients with IH and has only been investigated as a primary fascial closure technique to prevent IH in one previous randomized study^18^. In the Rein4CeTo1 trial^19^, RTL suture combined with the 4 : 1 small-bite technique was compared with the 4 : 1 small-bite technique alone in elective patients undergoing colorectal cancer (CRC) resection by midline laparotomy. The 1-year results of this trial showed that RTL with the 4 : 1 small-bite technique significantly reduced the incidence of computed tomography (CT)-diagnosed IH. However, it is well known that IHs develop over time and, although the short-term results of the present randomized clinical trial (RCT) favoured use of RTL sutures, the long-term effect of the technique is largely unknown. Hence, solid long-term data comparing RTL suture use with the current standard is needed to further explore its potential and usefulness in reducing the high incidence of IH, and justify widespread clinical implementation.

The aim of the present study was to evaluate the 3-year incidence of CT-diagnosed IH in the Rein4CeTo1 trial and investigate potential risk factors for IH.

## Methods

### Trial design

This was an open parallel RCT of CT-diagnosed IH incidence comparing two fascial closure techniques, in a 1 : 1 allocation ratio. The study included patients undergoing open CRC surgery at Skåne University Hospital Malmö and Kristianstad County Hospital, Sweden.

The trial complied with CONSORT guidelines. The Regional Ethics Committee of Lund University, Sweden (2017/459) approved the study, and it was registered at ClinicalTrials.gov (NCT03390764). The study protocol is available in the *supplementary material*. EuroQol (EuroQol Group, Rotterdam, the Netherlands) provided authorization for the research use of EQ-5D-5L™ (ID:21776).

### Participants

Patients aged > 18 years with CRC scheduled for resection by midline laparotomy between 2017 and 2021, were eligible for inclusion. Preoperative exclusion criteria were: cytoreductive surgery/hyperthermic intraperitoneal chemotherapy, previous midline hernia surgery, current midline hernia (umbilical hernia < 1 cm accepted), American Society of Anesthesiologists fitness grade > III, and inability to participate in follow-up. Perioperative exclusion criteria were: requirement for fascial reconstruction, previously unknown midline hernia > 1 cm, peritoneal carcinomatosis, and any inappropriate deviation from the planned surgery at the discretion of the managing surgeon. Patients undergoing reoperation for reasons other than IH repair, and those without confirmed IH at 1-year follow-up and who did not complete scheduled 3-year postoperative CT were excluded.

### Interventions

Patients were managed according to existing routines and randomized either to the RTL or PDS group during surgery just before fascial closure. Participating CRC surgeons carried out the fascial closure in accordance with instructions given by the study managers, as described previously^19^.

**Fig. 1:**
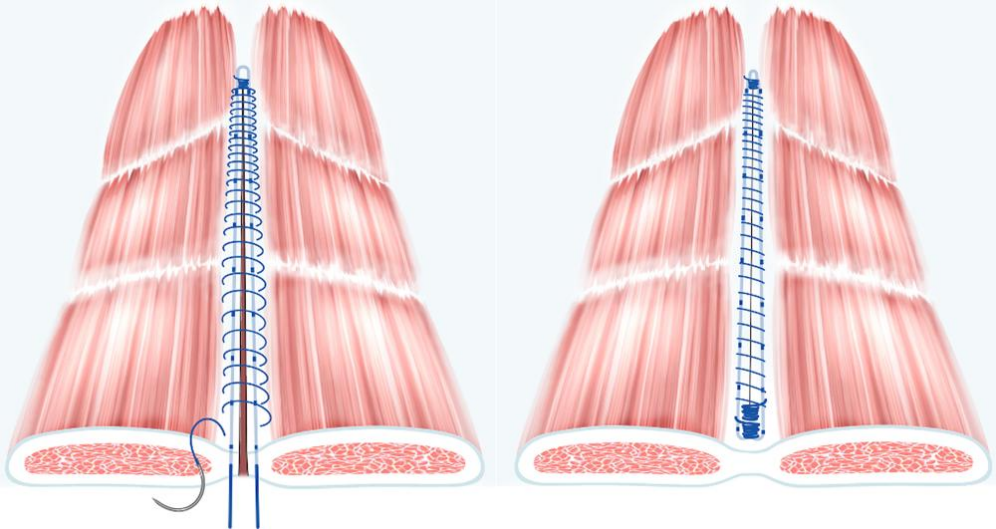
Schematic illustration of reinforced tension-line combined with 4 : 1 suture

The fascial incision in the RTL group was provided with a reinforcing (RTL) 2/0 polypropylene suture (Prolene^®^; Ethicon, Raritan, NJ, USA) on a CT-2 needle, threaded within the fascia on both sides, 5–8 mm from the incision, leaving the suture ends untied initially^17^. Accidental openings in the rectus muscle sheath were closed with the RTL suture. Subsequently, the incision was closed with the same type of suture (2/0 Prolene^®^), utilizing the 4 : 1 small-bite technique^10^. Sutures were placed 5 mm apart, just outside and including the RTL suture in each stitch. Finally, the RTL suture was tied (*Fig. 1***)**.

In the PDS group, the fascial incision was closed with a 2/0 polydioxanone suture (PDS^®^ Plus; Ethicon) on a CT-2 needle, 5–8 mm from the incision, 5 mm apart, according to the 4 : 1 small-bite technique used in the RTL group.

Skin closure was performed with a running intracutaneous 4/0 polydioxanone suture (PDS^®^ Plus; Ethicon) in both groups.

### Outcomes

The primary outcome of the Rein4CeTo1 RCT was incidence of CT-diagnosed IH at 1 year ± 3 months, and these results have been published previously^19^. The aim of the present study was to compare the long-term (36 ± 3 months) incidence of CT-diagnosed IH between the two study groups. Patients underwent CT without the Valsalva manoeuvre in the supine position, 1 and 3 years after surgery as part of routine CRC follow-up. CT images were reviewed independently by two surgeons and one radiologist, who were blinded to the fascial closure technique. Any discrepancies were discussed to reach consensus. IH was defined as any abdominal wall gap in a postoperative scar, perceptible or palpable by clinical examination or imaging, in accordance with the EHS definition. Umbilical hernias < 1 cm, present before and after surgery, were not considered as IHs.

Patients were invited to follow-up visits, including abdominal wall examinations in the relaxed state and when straining/coughing in both standing and supine positions, 1 and 3 years after surgery. Those not attending the 3-year visit were asked questions regarding bulging, discomfort, pain or any event possibly related to the operation, by letter or telephone. Patient data were registered prospectively, according to the study protocol, and clinical records were reviewed to identify events between visits.

### Sample size

Power computations in the Rein4CeTo1 trial were based on the primary outcome, CT-detected IH incidence at 1 year, amounting to a minimum of 90 randomized patients in each group with 80% power and 0.05 significance level, as described previously^19^. The study was not powered for analysis of any of the secondary outcomes, including the aim of the present report, CT-detected IH at 3 years.

### Randomization

Microsoft^®^ Excel 365 for Windows^®^ (Microsoft, Redmond, WA, USA) was used to generate the randomization sequence. The computer-generated list was used for allocation. The randomization was stratified by study centre with a 1 : 1 distribution between the study groups and block sizes of 4, 6 or 8. The group allocations were placed in sequentially numbered, sealed opaque envelopes.

### Blinding

Recruiting and operating surgeons were blinded to all aspects of the randomization process, and were unaware of block sizes. The three CT examiners were blinded regarding closure technique, as were the surgeons examining patients in the outpatient clinic.

### Statistical analysis

Continuous variables are expressed as mean(standard deviation). Potential risk factors for IH, comprising risk factors stated in guidelines (body mass index (BMI), diabetes, smoking, wound infection, and immunotherapy) as well as adjuvant chemotherapy and tumour location, specific for the present study, were analysed in univariate and bivariate logistic regression, with adjustment for fascial closure technique in the bivariate analyses. A multivariate logistic regression analysis was then performed, adjusting for closure technique with the three factors with the highest odds ratios (ORs) in the previous analysis; 95% confidence intervals were used when appropriate.

## Results

### Recruitment and participant flow

**Fig. 2:**
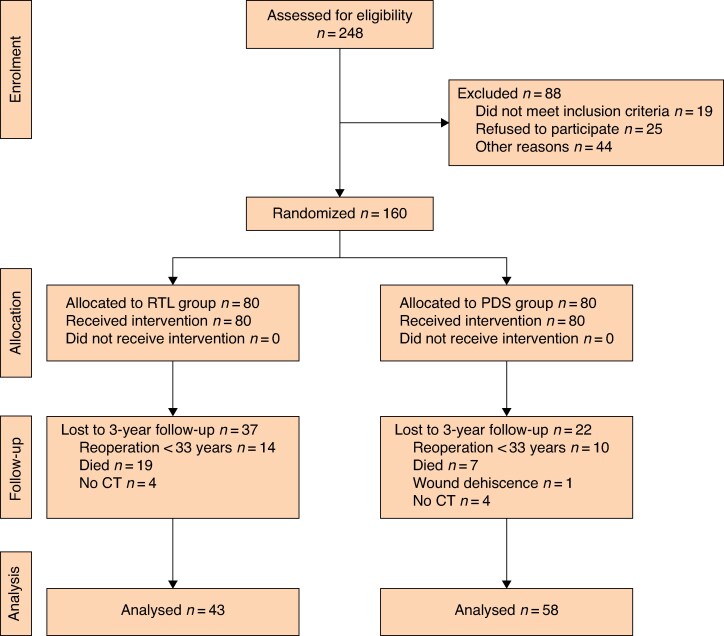
CONSORT diagram for the trial RTL, reinforced tension-line suture combined with 4 : 1 small-bite closure, performed with polypropylene; PDS, 4 : 1 small-bite closure alone with polydioxanone sutures; CT, computed tomography.

Between 2017 and 2021, a total of 248 patients were assessed as eligible for inclusion, of whom 134 remained for analysis at 1 year, reaching a power of 75.1% and not the desired 80%. Inclusion was stopped before reaching the intended study population because the COVID-19 pandemic led to organizational changes in the region, and an increase in minimally invasive surgery for CRC. Inclusion and exclusion are summarized in a CONSORT diagram (*Fig. 2*) and have been described in detail previously^19^. In total, 101 patients remained for analysis after 3 years (± 3 months): 43 in the RTL group and 58 in the PDS group, constituting the present study population (*Table 1*). Of the 101 patients, 75 attended a clinical follow-up visit after 3 years.

**Table 1:** Patient characteristics

	Randomized patients			Evaluable patients at 3 years*			Lost to follow-up
	Total (*n* = 160)	RTL (*n* = 80)	PDS (*n* = 80)	Total (*n* = 101)	RTL (*n* = 43)	PDS (*n* = 58)	Total (*n* = 59)
Age (years), mean(s.d.)	68(12)	70(11)	67(12)	69(10)	70(10)	68(11)	68(14)
**Sex**							
Male	86 (53.7%)	42 (52%)	44 (55%)	60 (59.4%)	26 (60%)	34 (59%)	26 (44%)
Female	74 (46.3%)	38 (48%)	36 (45%)	41 (40.6%)	17 (40%)	24 (41%)	33 (56%)
BMI (kg/m^2^), mean(s.d.)	25.6(4.3)	25.9(4.3)	25.4(4.4)	25.8(4.1)	25.9(4.0)	25.7(4.2)	25.4(4.7)
Previous midline incision	83 (51.9%)	38 (48%)	45 (56%)	57 (56.4%)	21 (49%)	36 (62%)	26 (44%)
Cardiovascular disease	80 (50.0%)	45 (56%)	35 (44%)	51 (51.0%)	24 (56%)	27 (47%)	29 (49%)
Chronic obstructive pulmonary disease	15 (9.4%)	8 (10%)	7 (9%)	10 (9.9%)	4 (9%)	6 (10%)	5 (9%)
Diabetes mellitus	23 (14.4%)	12 (15%)	11 (14%)	13 (12.9%)	7 (16%)	6 (10%)	10 (17%)
Steroid treatment	7 (4.4%)	4 (5%)	3 (4%)	2 (2.0%)	1 (2%)	1 (2%)	5 (9%)
Other immunosuppression	3 (1.9%)	1 (1%)	2 (3%)	1 (1.0%)	0 (0%)	1 (2%)	2 (3%)
**ASA fitness grade**							
I	13 (8.1%)	6 (8%)	7 (9%)	9 (8.9%)	3 (7%)	6 (10%)	4 (7%)
II	93 (58.1%)	42 (53%)	51 (64%)	66 (65.3%)	28 (65%)	38 (66%)	27 (46%)
III	54 (33.8%)	32 (40%)	22 (28%)	26 (25.7%)	12 (28%)	14 (24%)	28 (48%)
Smoker	20 (12.5%)	12 (15%)	8 (10%)	14 (13.9%)	7 (16%)	7 (12%)	6 (10%)
Haemoglobin (g/l), mean(s.d.)	124(13)	123(14)	124(12)	124(12)	124(13)	125(12)	123(15)
Albumin (g/l), mean (s.d.)	37(5)	37(5)	37(5)	38(4)	38(3)	37(5)	37(6)

Values are *n* (%) unless otherwise stated. *Including patients with incisional hernia during study period. RTL, reinforced tension-line suture combined with 4 : 1 small-bite closure, performed with polypropylene; PDS, 4 : 1 small-bite closure alone with polydioxanone sutures; s.d., standard deviation; BMI, body mass index; ASA, American Society of Anesthesiologists.

### Baseline data and surgical outcomes

Short-term (1-year) data including complications and IH rate have been published previously^19^. Demographic data for randomized patients, those remaining at 3-year follow-up, and patients lost to follow-up are presented in *Table 1*. Of patients available for 3-year follow-up, adjuvant chemotherapy was administered to 44 of 101 (43.6%): 17 of 43 (40%) in the RTL and 27 of 58 (47%) in the PDS group. Some 43 of 101 resections (42.6%) were colonic, 53 of 101 (52.5%) rectal, and 5 of 101 (5.0%) were pelvic exenterations. In total, 51 of 101 patients (50.5%) received a stoma: 25 of 43 (58%) in the RTL group and 26 of 58 (45%) in the PDS group. Among the 101 patients, a suture : wound length ratio > 4 was achieved in all but 2, both in the PDS group. Data on suture length ratio were missing for four patients, two in each group.

### Outcomes and analysis

Of the 160 randomized patients, 1 in the PDS group developed wound dehiscence. Within 3 years, 24 patients underwent reoperations for reasons other than IH, 26 died, and 8 patients did not undergo CT at 3 years (*Fig. 2*).

**Fig. 3:**
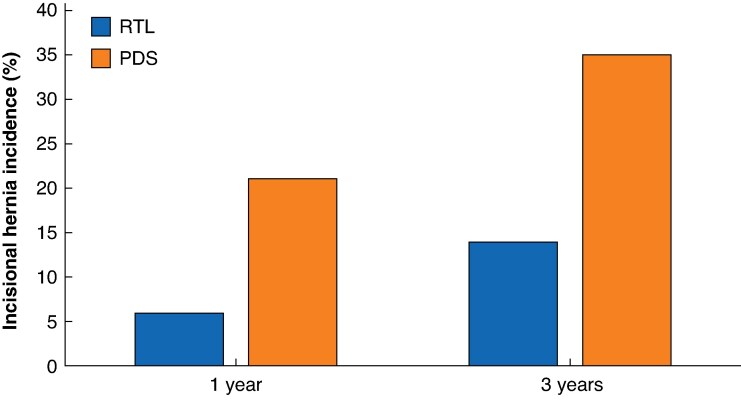
Incisional hernia incidence at 1 and 3 years RTL, reinforced tension-line suture combined with 4 : 1 small-bite closure, performed with polypropylene; PDS, 4 : 1 small-bite closure alone with polydioxanone sutures.

The incidence of IH per protocol 1 year after surgery was 19 of 134 (14.2%), as reported previously^19^. After a further 2 years, eight new IHs had occurred and no patients with known IH were lost to follow-up at 3 years. At 3 years, 101 patients remained for follow-up, and the cumulative incidence of CT-diagnosed IH in per-protocol analysis was 27 of 101, with a statistically significant difference between the groups: 6 of 43 (14%) in the RTL group and 21 of 58 (36%) in the PDS group; the risk difference was 22% (OR 3.50, 95% confidence interval (c.i.) 1.27 to 9.66; *P* = 0.016) (*Fig. 3*). The IH rate was similar in the two operating centres (OR 1.11, 0.32 to 3.90; *P* = 0.867). The incidence of IH according to intention-to-treat analysis was 27 of 160 (16.9%): 6 of 80 (8%) in the RTL group and 21 of 80 (26%) in the PDS group, without imputation for patients lost to follow up. Of the 27 incisional hernias, 8 were detected between 1 and 3 years, 2 in the RTL group and 6 in the PDS group. The width of the hernias was < 4 cm in 11 patients, 4–10 cm in 14 patients, and > 10 cm in 2 patients.

### Clinical examination and IH surgery

Of the 27 patients with IH, 21 attended 1- or 3-year clinical visits. In total, 6 of 21 CT-confirmed hernias in these patients were missed on clinical examination, 5 hernias < 4 cm in size and 1 defect of 4–10 cm. IH surgery was undertaken in two patients between the 1- and 3-year follow-up. In addition, one patient was scheduled for IH surgery but died from metastatic disease before the operation, one is awaiting surgery, four were reluctant to have surgery, and one with minor symptoms has currently not been recommended for surgery.

### Adjustment for and investigating potential risk factors for IH

The incidence of IH according to co-variates and potential risk factors can be seen in *Table 2*. Univariate and bivariate logistic regression analyses were undertaken for the following potential risk factors: diabetes, smoking, obesity (BMI > 30 kg/m^2^), wound infection, tumour location, and adjuvant chemotherapy (*Table 3*). Steroid treatment is also listed as a risk factor for IH in guidelines, but was not included in the analysis because only two patients had this medication. The incidence of IH was 63% among patients who developed a wound infection, 53% in those with a BMI > 30 kg/m^2^, and 41% in patients who received adjuvant chemotherapy (*Table 2*). After adjusting for study group, all three factors were significantly associated with IH in univariate and bivariate analyses (*Table 3*). In multivariate analysis adjusting study groups for these factors, the difference between the RTL and PDS groups remained statistically significant (OR 3.40, 95% c.i. 1.14 to 10.14; *P* = 0.028) (*Table 4*). Besides fascial closure with the 4 : 1 small-bite technique and polydioxanone sutures (PDS group), adjuvant chemotherapy was the only other significant independent risk factor for IH (OR 2.98, 1.10 to 8.08; *P* = 0.032) (*Table 4*).

**Table 2:** Potential risk factors for IH within 3 years

	IH prevalence
**Sex**	
Female	9 of 41 (22%)
Male	18 of 60 (30%)
**Age (years)**	
≥ 70	15 of 55 (27%)
< 70	12 of 46 (26%)
**BMI (kg/m^2^)**	
≥ 30	8 of 15 (53%)
< 30	19 of 86 (22%)
**Smoker**	
Yes	2 of 14 (14%)
No	25 of 87 (29%)
**Chronic obstructive pulmonary disease/asthma**	
Yes	3 of 10 (30%)
No	24 of 91 (26%)
**Diabetes**	
Yes	3 of 13 (23%)
No	24 of 88 (27%)
**Cardiovascular disease**	
Yes	11 of 51 (22%)
No	16 of 50 (32%)
**ASA fitness grade**	
I–II	22 of 75 (29%)
III–V	5 of 26 (19%)
**Steroid treatment**	
Yes	2 of 2 (100%)
No	25 of 99 (25%)
**Neoadjuvant cytostatic**	
Yes	11 of 42 (26%)
No	16 of 59 (27%)
**Previous midline operation**	
Yes	14 of 57 (25%)
No	13 of 44 (30%)
**Previous stoma**	
Yes	8 of 29 (28%)
No	19 of 72 (26%)
**New stoma**	
Yes	12 of 51 (24%)
No	15 of 50 (30%)
**Stoma closure**	
Yes	4 of 18 (22%)
No	23 of 83 (28%)
**Tumour location**	
Rectum	12 of 58 (21%)
Colon	15 of 43 (35%)
**Accidental opening of rectus sheath above or around umbilicus**	
Yes	10 of 40 (25%)
No	15 of 59 (25%)
**Suture : wound length ratio**	
> 4	24 of 95 (25%)
< 4	1 of 2 (50%)
**Duration of operation (minutes)**	
≥ 380	14 of 51 (28%)
< 380	13 of 50 (26%)
**Perioperative complication**	
Yes	0 of 7 (0%)
No	27 of 94 (29%)
**Wound infection**	
Yes	5 of 8 (63%)
No	22 of 93 (24%)
**Complication within 30 days**	
Yes	17 of 63 (27%)
No	10 of 38 (26%)
**Clavien–Dindo complication grade**	
0−II	25 of 90 (28%)
III	2 of 11 (18%)
**Adjuvant chemotherapy**	
Yes	18 of 44 (41%)
No	9 of 56 (16%)

Values are *n* (%). IH, incisional hernia; BMI, body mass index; ASA, American Society of Anesthesiologists.

**Table 3:** Univariate logistic regression of potential risk factors for incisional hernia, adjusted for fascial closure technique in bivariate analysis

	*n*	Univariate analysis		Bivariate analysis*	
		Odds ratio	*P*	Odds ratio	*P*
**BMI > 30 kg/m^2^**					
No	86	1.00 (reference)		1.00 (reference)	
Yes	15	4.03 (1.30, 12.54)	0.016	3.55 (1.10, 11.45)	0.034
**Smoker**					
No	87	1.00 (reference)		1.00 (reference)	
Yes	14	0.41 (0.09, 1.98)	0.269	0.44 (0.09, 2.17)	0.310
**Diabetes**					
No	88	1.00 (reference)		1.00 (reference)	
Yes	13	0.80 (0.20, 3.16)	0.750	0.93 (0.22, 3.84)	0.915
**Tumour location**					
Colon	43	1.00 (reference)		1.00 (reference)	
Rectum	58	0.49 (0.20, 1.19)	0.114	0.62 (0.24, 1.58)	0.317
**Wound infection**					
No	93	1.00 (reference)		1.00 (reference)	
Yes	8	5.38 (1.19, 24.33)	0.029	5.82 (1.19, 28.56)	0.030
**Adjuvant chemotherapy**					
No	56	1.00 (reference)		1.00 (reference)	
Yes	44	3.62 (1.42, 9.19)	0.007	3.58 (1.37, 9.38)	0.009

Values in parentheses are 95% confidence intervals. *Adjusted for study group. BMI, body mass index.

**Table 4:** Univariate and multivariate regression analysis of potential risk factors for incisional hernia

	*n*	Univariate analysis		Multivariate analysis*	
		Odds ratio	*P*	Odds ratio	*P*
**Fascial closure group**					
RTL	43	1.00 (reference)		1.00 (reference)	
PDS	58	3.50 (1.27, 9.66)	0.016	3.40 (1.14, 10.14)	0.028
**Adjuvant chemotherapy**					
No	56	1.00 (reference)		1.00 (reference)	
Yes	44	3.62 (1.42, 9.19)	0.007	2.98 (1.10, 8.08)	0.032
**BMI (kg/m^2^)**					
< 30	86	1.00 (reference)		1.00 (reference)	
≥ 30	15	4.03 (1.30, 12.54)	0.016	2.69 (0.76, 9.50)	0.124
**Wound infection**					
No	93	1.00 (reference)		1.00 (reference)	
Yes	8	5.38 (1.19, 24.33)	0.029	3.66 (0.66, 20.24)	0.136

Values in parentheses are 95% confidence intervals. *Includes all four variables. RTL, reinforced tension-line suture combined with 4 : 1 small-bite closure, performed with polypropylene; PDS, 4 : 1 small-bite closure alone with polydioxanone sutures; BMI, body mass index.

### Harms

Complication rates and unintended effects were similar in the RTL and PDS groups.

## Discussion

The present two-centre RCT has evaluated 3-year IH incidence, comparing use of RTL suture and the 4 : 1 small-bite technique with the standard 4 : 1 small-bite technique, in patients undergoing elective CRC surgery with a midline laparotomy. The study has shown that use of the RTL suture with 4 : 1 small-bite technique significantly reduces the long-term incidence of IH, compared with use of the 4 : 1 small-bite technique alone. In fact, the incidence of IH in the RTL group was less than half of that in the PDS group, and the difference was statistically significant after adjustment for potential confounding factors. In addition, adjuvant chemotherapy was found to be a strong and independent risk factor for IH.

The early results of the Rein4CeTo1 trial indicated that the incidence of IH was 14.2% (19 of 134) 1 year after surgery^19^. With eight additional IHs diagnosed between 1 and 3 years after operation, the overall CT-detected cumulative 3-year IH incidence was 26.7%, with a significant difference between the RTL (14.0%) and PDS (36.2%) groups. Notably, the incidence in the PDS group was substantially higher than in three previous RCTs^10,12,20^, which reported IH rates of 5.6–13.0% for the small-bite technique. However, two of these studies^10,12^ reported short-term results (after 1 year) and IHs are known to develop over time. In fact, the incidence of IH increased from 6 to 14% in the RTL group and from 21 to 36% in the PDS group, between 1 and 3 years, respectively. In addition, CT was used to detect IH in the present study, in contrast to the aforementioned studies^10,12,20^ that used clinical examination alone, or combined with ultrasonography or CT in selected patients. Thus, IH can be missed on clinical examination, as exemplified by 6 of 21 CT-detected IHs being missed on clinical examination here.

Another explanation for the discrepancy in IH incidence between the present study and the literature is patient selection. The present study included patients undergoing surgery for CRC, which is classified as a clean-contaminated procedure, and patients were in general elderly with co-morbidities; in contrast, previous studies included a wide range of surgical patients and one^20^ even excluded patients at high risk of IH (BMI > 30 kg/m^2^, neoadjuvant treatment, immunosuppression). In this context, it is also important to note that the present study mainly reports the results of per-protocol analysis, and the IH incidence was 16.9% (27 of 160) when calculated by intention-to-treat analysis, although without imputation for patients lost to follow-up.

Moreover, use of a RTL suture has only been evaluated as a prophylactic technique to prevent IH in one previous RCT^18^, which compared RTL with the 4 : 1 technique in emergency and elective midline laparotomies in patients considered to be at high risk for IH. That study reported a 3-year IH incidence of 9.8% with the RTL technique *versus* 28.3% with the 4 : 1 technique, in line with the present findings. Nevertheless, it did not report cumulative IH incidence and patients with previous laparotomies were excluded, in contrast to the present study.

Non-absorbable polypropylene sutures were used in the RTL group in the present study because the technique is intended to provide a permanent reinforcement. In contrast, absorbable polydioxanone was used in the small-bite 4 : 1 PDS group, as this is the standard technique and recommended by guidelines. Use of different suture materials could be argued to be the reason for the difference in IH and not the suturing technique itself. However, a previous large meta-analysis^21^ showed no difference in IH rate between use of absorbable and non-absorbable sutures. In addition, a previous biomechanical study^22^ of human cadavers found that tissue tensile strength was 31% higher with the RTL suture compared with conventional sutures, supporting the notion that the RTL technique itself reduces the IH rate, as shown here.

Use of prophylactic mesh is an alternative to the 4 : 1 small-bite technique to prevent IH in high-risk patients, according to guidelines^23^. Interestingly, a recent meta-analysis^24^ showed that the incidence of IH was 13.4% with prophylactic mesh, which is very similar to IH incidence with the RTL technique in the present study. In addition, implementation of prophylactic mesh is hampered by an increased risk of complications, time consumption, and increased costs^13–16^. In the present study, there was no increase in complications in the RTL group, the number needed to treat was 4.9, and the operating time was increased by only 12 minutes, compared with that in the PDS group^19^.

Risk factors for developing IH have been well studied and current guidelines^25^ state that overweight or obesity (BMI ≥ 25 kg/m^2^), wound infection, immunosuppression, smoking, and diabetes are important risk factors to consider. In the present study, univariate logistic regression analysis was undertaken to identify risk factors for IH, and adjusted for fascial closure technique in bivariate analysis. Obesity (BMI > 30 kg/m^2^), wound infection, and adjuvant chemotherapy were significant risk factors in univariate and bivariate analyses. Indeed, high BMI and wound infections are well known risk factors for IH, but adjuvant chemotherapy has not been reported as a risk factor for IH previously. Notably, adjuvant chemotherapy was also a significant risk factor in the multivariate analysis, adjusting for study group, BMI, and wound infection. An explanation for this finding could be that adjuvant chemotherapy has a negative effect on wound healing and thereby leads to an increased risk of IH. Alternatively, adjuvant chemotherapy is a proxy for more advanced and metastasized CRC, which possibly has impaired wound healing abilities. Smoking and diabetes are also well known risk factors for IH but were not significant in the present logistic regression analyses. However, this study was not methodologically designed or powered for risk factor analysis, and the relatively small number of included patients limits any firm conclusions because of an apparent risk of type II errors. Importantly, when adjusting study groups with the three strongest risk factors in multivariate analysis (BMI > 30 kg/m^2^, wound infection, and adjuvant chemotherapy), the PDS group remained a statistically significant and strong risk factor for IH. Thus, the difference in IH incidence between the RTL and PDS group is independent of these co-existing risk factors.

The implementation of minimally invasive techniques in surgery in general, and CRC surgery in particular, has increased dramatically during the past decades. Recruitment to the present study was stopped earlier than planned partly owing to a decrease in midline laparotomies for CRC surgery in favour of laparoscopic or robotic surgery. However, although minimally invasive techniques are becoming the new standard for CRC surgery, midline laparotomy is still and will continue to be used in patients who are not suitable for minimally invasive surgery. In addition, a limited incision is needed for specimen extraction and, although the laparotomy is limited, the risk of IH remains. In fact, the incidence of IH has been reported to be as high as 16% following limited midline incision for specimen extraction in CRC surgery^7^. In addition, a recent study^26^ investigating IH in patients with CRC found no difference in IH incidence after midline laparotomy *versus* laparoscopy with specimen extraction. Importantly, although open midline laparotomy for CRC surgery was chosen as the setting in the present study, the generalizability of the findings to other midline laparotomies and limited tissue extraction laparotomies is thought to be high.

This study is strengthened by the randomized design, two recruiting centres, few missing data, and good adherence to the protocol. It is further empowered by including univariate, bivariate, and multivariate analyses, adjusting for potential confounding factors. However, there are certain limitations. First, the study was powered for the primary aim, CT-diagnosed IH incidence at 1 year and not for the aim of the present study, incidence of CT-diagnosed IH at 3 years. Second, the study was terminated before reaching the intended number of patients owing to a decrease in midline laparotomies in favour of minimally invasive techniques, and the COVID-19 pandemic altering patient flow in the region. In addition, a significant portion of included patients was lost to follow-up, also in part because of travelling restrictions during the pandemic.

This study has demonstrated that the RTL with a 4 : 1 small-bite technique significantly reduces the long-term incidence of IH compared with the 4 : 1 small-bite technique alone in patients undergoing open surgery for CRC. These findings suggest that adding an RTL suture is an efficient way to prevent a considerable number of IHs, without increasing costs and risk of complications, and only marginally increasing operating time. Possible advantages need to be evaluated in other patient cohorts and situations.

## Supplementary Material

zraf150_Supplementary_Data

## Data Availability

Study data are available on request.
